# Free-living gait does not differentiate chronic mTBI patients compared to healthy controls

**DOI:** 10.1186/s12984-022-01030-6

**Published:** 2022-05-26

**Authors:** Dylan Powell, Alan Godfrey, Lucy Parrington, Kody R. Campbell, Laurie A. King, Sam Stuart

**Affiliations:** 1 Department of Computer and Information Sciences, Northumbria University, Newcastle-upon-Tyne, UK; 2 Department of Neurology, Oregon Health and Science University, Portland, OR USA; 3 Department of Dietetics, Human Nutrition and Sport, La Trobe University, Victoria, Australia; 4 Department of Sport, Exercise and Rehabilitation, Northumbria University, Newcastle-upon-Tyne, NE1 8ST UK; 5 North Tyneside Hospital, Northumbria Healthcare NHS Foundation Trust, North Shields, UK

**Keywords:** mTBI, Concussion, Inertial measurement unit, Gait

## Abstract

**Background:**

Physical function remains a crucial component of mild traumatic brain injury (mTBI) assessment and recovery. Traditional approaches to assess mTBI lack sensitivity to detect subtle deficits post-injury, which can impact a patient’s quality of life, daily function and can lead to chronic issues. Inertial measurement units (IMU) provide an opportunity for objective assessment of physical function and can be used in any environment. A single waist worn IMU has the potential to provide broad/macro *quantity* characteristics to estimate gait mobility, as well as more high-resolution micro spatial or temporal gait characteristics (herein, we refer to these as measures of *quality*). Our recent work showed that quantity measures of mobility were less sensitive than measures of turning quality when comparing the free-living physical function of chronic mTBI patients and healthy controls. However, no studies have examined whether measures of gait quality in free-living conditions can differentiate chronic mTBI patients and healthy controls. This study aimed to determine whether measures of free-living gait quality can differentiate chronic mTBI patients from controls.

**Methods:**

Thirty-two patients with chronic self-reported balance symptoms after mTBI (age: 40.88 ± 11.78 years, median days post-injury: 440.68 days) and 23 healthy controls (age: 48.56 ± 22.56 years) were assessed for ~ 7 days using a single IMU at the waist on a belt. Free-living gait quality metrics were evaluated for chronic mTBI patients and controls using multi-variate analysis. Receiver operating characteristics (ROC) and Area Under the Curve (AUC) analysis were used to determine outcome sensitivity to chronic mTBI.

**Results:**

Free-living gait quality metrics were not different between chronic mTBI patients and controls (all p > 0.05) whilst controlling for age and sex. ROC and AUC analysis showed stride length (0.63) was the most sensitive measure for differentiating chronic mTBI patients from controls.

**Conclusions:**

Our results show that gait quality metrics determined through a free-living assessment were not significantly different between chronic mTBI patients and controls. These results suggest that measures of free-living gait quality were not impaired in our chronic mTBI patients, and/or, that the metrics chosen were not sensitive enough to detect subtle impairments in our sample.

## Introduction

Traumatic brain injuries (TBI) can be broadly defined as sudden trauma causing damage to the brain, with severity ranging from mild TBI (mTBI; commonly known as concussion) to severe TBI [1]. An array of impairments accompany TBI, such as deficits in physical (balance, gait and turning) [2, 3], psychological (cognitive impairments and symptoms) [4], and sensory function (visual or vestibular deficits) [5]. Such deficits can be subtle and difficult to detect in mTBI and may persist for long periods after the initial injury (e.g., > 3 months). Chronic symptoms post-mTBI can significantly impact quality of life and daily function, which can lead to prolonged issues/symptoms [6]. Physical impairments are especially prevalent in mTBI, with eight out of ten people with acute mTBI reporting balance impairments within a few days of the injury and three out of ten reporting longer-term (chronic) balance or gait impairments [5, 7, 8]. Therefore, physical testing (balance and gait) remains a crucial component of clinical assessment to quantify impairment across various mTBI timelines [9–12]. Understanding gait and balance deficits may provide targets for rehabilitation.

Balance impairment is commonly assessed in the acute stage following mTBI [13, 14], primarily using the Balance Error Scoring System (BESS). The BESS requires a clinician to manually record errors each time the patient fails to maintain a balance stance position. However, the sensitivity of the BESS is highly variable due to considerable subjectivity in error counting, which impacts the replicability and validity of results [15–18]. Additionally, subtle balance deficits may be visually undetectable by a clinician’s subjective assessment and therefore unmeasurable. Other physical impairments, such as gait deficits, are often not examined by clinicians following acute mTBI. Tandem gait/walking may be done as part of the Sports Concussion Assessment Tool (SCAT), however clinician observation has been found to miss subtle gait deficits that persist in chronic mTBI patients (i.e. due to low ceiling effect of the test) [19]. To detect subtle gait deficits following mTBI, assessment is typically conducted in research settings with objective laboratory equipment, such as force plates and 3D motion capture [7, 20–23]. As such, there have been improvements in objective and instrumented assessment which can yield greater sensitivity than traditional qualitative methods of assessment [14].

Results from laboratory-based objective gait assessment have found pace-related deficits (stride length and gait speed) in chronic mTBI patients compared with healthy controls [24], suggesting gait may be a useful diagnostic marker of mTBI. While laboratory studies provide a foundation for evaluating the differences between healthy and impaired gait, laboratory-centric assessment methods are prescriptive in nature, and may mask subtle mTBI-related deficits that may otherwise occur within habitual (free-living) environments. Accordingly, monitoring gait beyond the laboratory may provide an opportunity to detect subtle and meaningful deficits following mTBI.

Continuous gait monitoring in free-living environments is becoming more common, due to the widespread use of discrete inertial-based measurement units (IMU), which are the accepted standard for gathering continuous, high-resolution data [25, 26]. IMUs can estimate general mobility outcomes (e.g. measures of quantity such as steps per day) or more refined balance, gait and turning outcomes characterising quality of movement within any environment (e.g. stride length or turning speeds) [2, 14, 27–30]. Our recent work examined free-living mobility quantity and turning quality measures in chronic mTBI patients and controls. We found turning quality metrics to be more sensitive than mobility quantity metrics to differentiate groups [3]. Specifically, those with chronic mTBI had larger, slower and more variable turns during daily life, but had a similar number of steps per day compared with controls [3]. While that study evaluated turning quality, it did not measure other gait quality metrics such as stride velocity, step length, or swing time. Additionally, while previous studies have examined mTBI gait in research settings, no study to date has comprehensively quantified free-living gait quality in chronic mTBI patients and healthy controls. Therefore, a gap remains as to whether measures of free-living gait quality are impaired in chronic mTBI patients. Greater understanding of how mobility is affected in free-living environments may uncover useful markers for subtle deficits in chronic mTBI patients.

The aims of this study were therefore to; (1) explore if free-living gait is impaired in people with chronic mTBI compared with healthy controls, and (2) determine the most sensitive free-living gait quality metrics that differentiate chronic mTBI patients from controls. We hypothesise that free-living mobility would be impaired in chronic mTBI patients compared to controls, with selective gait quality characteristics sensitive to differentiate chronic mTBI.

## Methods

### Participants

Thirty-two symptomatic chronic mTBI patients and 23 healthy controls participated. Participants were recruited as part of a larger study [31], through posters in athletic facilities, physical therapy clinics, hospitals, concussion clinics, community notice boards, and cafes in and around the Portland, OR metropolitan area. Patient demographics are shown in Table 1. Ethical approval was granted by the Oregon Health and Science University (OHSU) and Veterans Affairs Portland Health Care System (VAPORHCS) joint institutional review board with participants providing written informed consent before commencing the study.

**Table 1:** Participant demographics

	Controls (n = 23)	mTBI (n = 32)	p
Age (years)	48.56 (22.56)	40.88 (11.78)	0.11
Sex (male or female) ^b^	M(6) F(17)	M(6) F(26)	0.52
Height (cm)	165.46 (8.03)	168.51 (9.19)	0.22
Mass (kg)	68.03 (15.32)	76.17 (18.80)	0.25
NSI total score	-	35.88 (13.9)	-
NSI vestibular	-	5.44 (2.22)	-
NSI somatosensory	-	10 (4.92)	-
NSI cognitive score	-	8.34 (3.89)	-
NSI affective score	-	10.34 (5.64)	-
Days since injury^a^	-	440.68 (700.63)	-

^a^Median and interquartile range

^b^chi-squared, Mean and standard deviation reported unless otherwise stated. mTBI, mild traumatic brain injury; NSI—neurobehavioral symptom inventory

### Inclusion and exclusion criteria

Participants were included in the chronic mTBI group if they had had a diagnosis of mTBI based upon Veteran Health Administration (VHA)/Department of Defense (DoD) [32] criteria and who were greater than three months post mTBI with self-reported balance impairments. The control group consisted of those who had no history of brain injury in the last year. Additionally, mTBI patients were required to have minimal to no cognitive deficits as determined by the Short-Blessed Test (score ≤ 8) [33] and no peripheral vestibular or oculomotor pathology preceding their mTBI. Participants were excluded if they had any musculoskeletal injury which could impair their gait or balance or a recent history of moderate or severe substance abuse.

### Gait analysis

Participants were asked to wear an IMU for 7 days, and participants with less than 3 days were excluded from analysis, in line with previous studies [3, 34, 35]. Participants wore a compact (L × W × H: 43.7 × 39.7 × 13.7 mm, 128 Hz) and lightweight (< 25 g) IMU (previously validated [36–38]) attached to a belt (128 Hz, Opal V1, APDM Inc., Portland, OR) that contained an accelerometer (± 16 g, ± 200 g) and gyroscope (± 2000 deg/s). Participants wore the IMU around their waist for a minimum of 5 h per day for up to 7 days using the protocol described previously by Fino et al. 2017 [31] and Stuart et al. 2020 [3]. Data were stored on the IMU internal storage (8 Gb) and then downloaded via proprietary software (MobilityLab, APDM Inc., Portland, OR) to a laptop. Free-living data were then processed using custom-made and validated MATLAB® (MathWorks Inc, Massachusetts, USA) algorithms to estimate 12 free-living gait quality metrics [34, 35, 39, 40].

#### Gait

Free-living measures of gait quality were calculated using a bespoke MATLAB® algorithm as follows. The waist worn IMU was used to examine orientation and periods of static and dynamic activity [39, 40]. Subsequently, the latter were examined for initial and final foot contact events within the gait cycle via the continuous wavelet transform [41], where a bout/period of walking was predefined by a time period of between 0.25 and 2.25 s and ≥ 3 steps [42]. For the purposes of this study, a movement bout was classified as > 10 s. Gait quality metrics included mean; stance time (seconds, s), step time(s), stride time (s), swing time (s), stride length (centimetres, cm), stride velocity (cm/second, *cms*^*−1*^*)* and coefficient of variation (CV) of these measures.

### Self-reported symptoms

Chronic mTBI patients completed the Neurobehavioral Symptom Inventory (NSI) which is widely used in the assessment of mTBI symptoms [24, 43]. The NSI is composed of 22 items within the questionnaire and recorded on a five-point Likert scale, with higher scores indicating more severe symptoms. The maximum a participant can score is 88. The NSI and subscales [44] have acceptable reliability in characterising presence and tracking severity of symptoms in TBI [44, 45]. The NSI remains the cornerstone of clinical symptom assessment and was determined as the appropriate method to capture self-reported impairments in the chronic mTBI patients.

### Statistical analysis

Data were analysed in SPSS (v23, IBM) and R studio (Boston, MA, USA). All data were normally distributed as assessed with Shapiro-Wilks tests and therefore parametric tests were used. Independent t-tests were performed comparing demographic information between mTBI and control groups. To compare free-living gait quality metrics between chronic mTBI patients and controls, we used separate multivariate analysis of covariance (MANCOVA). MANCOVA was used to control for sex and age [4, 46].

To estimate which gait quality metrics differentiated chronic mTBI patients from controls, we used receiver operating characteristic (ROC) and area under the curve (AUC) analysis. ROC analysis provides a trade-off between specificity and sensitivity between the various free-living gait quality metrics and binary classification of either mTBI patients and healthy control. Statistical significance was determined at *p* < *0.05 (two-tailed)* unless otherwise stated. Bonferroni corrected significance values were applied for multiple comparisons in free-living gait quality measures (*p* < *0.002).* Effect sizes were interpreted as small *(0.01)*, medium (*0.06*), and large *(0.14)* as previously described [47].

## Results

### Demographics and clinical assessments

Demographic characteristics are presented in Table 1 for age (years), height (cm), mass (kg) and the number of days since injury and NSI for the mTBI group only. In our mTBI cohort, NSI total score was moderately high (5th to 9th percentile) compared to previously published normative mTBI scores, demonstrating that our chronic mTBI group was still symptomatic at least more than 3 months after injury [44].

### Adherence to IMU device

Participants were asked to wear the IMU-based device for 7 days, but compliance was variable across both groups with several mTBI (n = 16) and control (n = 13) participants wearing the sensor for less than 7 days. Specifically, the mean number of days that the IMU was worn was 6.8 (± 2.4) days in the mTBI group and 6.04 (± 2.0) days in the control group.

### Group differences in free-living gait quality measures

When controlling for age and sex, there were no significant differences in measures of free-living gait quality between chronic mTBI patients (*p* > *0.05*) and controls. Descriptive data for free-living gait quality metrics are provided in Table 2.

**Table 2:** Free-living gait quality metrics; group differences whilst controlling for age and sex, Area under the Curve (AUC)

Free-living gait metric	mTBI (n = 32) mean (S.D.)	Controls (n = 23) mean (S.D.)	F	p	η_p_^2^	AUC
Mean stance time (seconds, s)	0.83 (0.05)	0.85 (0.09)	0.19	0.66	0.00	0.44
Mean step time (s)	0.70 (0.05)	0.73 (0.09)	0.21	0.65	0.00	0.44
Mean stride time *(s)*	1.41 (0.10)	1.45 (0.18)	0.21	0.65	0.00	0.44
Mean swing time (s)	0.58 (0.05)	0.60 (0.09)	0.22	0.64	0.00	0.44
Mean stride length (centimetres, cm)	74.01 (4.10)	72.68 (3.60)	2.84	0.10	0.05	***0.63***
Mean stride velocity (cms^−1^)	105.59 (8.88)	101.34 (11.47)	1.37	0.25	0.03	***0.60***
Stance time variability CV (s)	0.20 (0.01)	0.21 (0.02)	0.03	0.87	0.00	0.49
Step time variability CV (s)	0.20 (0.01)	0.20 (0.02)	0.10	0.75	0.00	0.48
Stride time variability CV (s)	0.22 (0.01)	0.22 (0.01)	0.35	0.56	0.01	***0.51***
Swing time variability CV (s)	0.20 (0.01)	0.21 (0.02)	0.13	0.72	0.00	*0.47*
Step length variability CV (s)	18.62 (1.18)	18.32 (0.96)	2.30	0.14	0.04	***0.61***
Step velocity variability CV (cms^−1^)	36.90 (3.11)	35.48 (4.08)	1.18	0.28	0.02	***0.60***

Bolded p values; p < 0.05 (Bonferroni corrected p value 0.002). Group analysis of covariance results controlling for age and sex. mTBI, mild traumatic brain injury; S.D., standard deviation; CV, coefficient of variation, η_p_^2^ partial eta squared of effect size, F Wilks’ λ,

AUC > 0.50 in italics and bold

### Sensitivity and specificity of free-living gait metrics

**Fig. 1:**
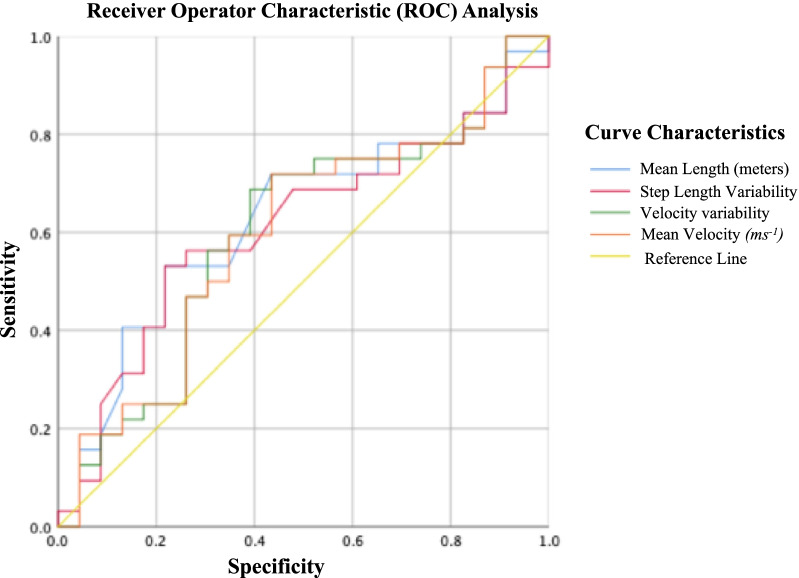
Receiver operator character (ROC) analysis for the top gait quality metrics (AUC > 0.51)

Figure 1 shows the receiver operating characteristics (ROC) analysis for the top four gait quality metrics (AUC > 0.51). Free-living gait quality (mean AUC: 0.51) was considered poor at differentiating chronic mTBI patients from controls (AUC > 0.50, Table 2).

## Discussion

This study progresses our previous work [3], which examined free-living activity quantity and turning quality measured by a single IMU in those with chronic mTBI compared to healthy controls. Free-living mobility assessment in mTBI is still an emerging research area, but results from other neurological conditions (e.g. Parkinson's disease) suggest that impaired gait occurs in parallel with neurological dysfunction [48]. However, results in this study indicated that free-living gait quality was not significantly different between our samples of chronic mTBI patients and healthy controls (when controlling for age and gender). The absence of significant differences in this study are likely multifactorial and could involve both inherent limitations of self-reporting of balance issues, and the chronicity of this mTBI cohort. However, assessment of free-living mobility in chronic mTBI may still allow for improved diagnostics and monitoring of recovery within real-world environments, which is unachievable using analog (non-digital) approaches or laboratory-based assessments only, but further research with longitudinal assessments following the initial injury would be required.

### Free-living gait quality measures are not impaired in chronic mTBI patients

Our results show that free-living gait quality metrics were not different between chronic mTBI and control groups, which is surprising given this cohort had self-reported balance deficits. Overall research into chronic mTBI has yet to gain consensus on what specific measures can differentiate healthy people from those with mTBI [24]. Indeed some laboratory-based studies have found pace-related deficits (stride length and gait speed) while other studies have found no differences outside of the acute timeframe (> 10 days) [2]. Laboratory gait assessment does allow for more controlled assessment of complex tasks (e.g. dual-task, obstacle avoidance, etc.), which may be required to elicit or provoke gait deficits in chronic mTBI [2, 49]. For example, dual-task laboratory assessment in people with chronic mTBI can reveal gait deficits in rhythm (stride time) [24]. However, complex laboratory tasks fail to fully replicate free-living environments where motor, cognitive and sensory function are continuously challenged [50]. Given these challenges in free-living environments, we were surprised that our measures of gait quality did not suggest impaired mobility in this chronic mTBI cohort.

The lack of significant differences and low effect sizes in gait quality measures between chronic mTBI patients and healthy controls may be related to the considerable chronicity (median 1.2 years post-injury) of this mTBI cohort. This duration may have resulted in the cohort developing chronic compensatory strategies over time to replicate ‘normal’ gait patterns during walking in their daily life. To fully understand this, future research should test participants in both the laboratory under complex conditions (e.g., dual-task, obstacle walking, turns course etc.) and in free-living environments longitudinally from the time of initial injury to better understand how gait changes acutely after mTBI and into more chronic stages. Similarly incorporating assessment of turning, which is a more complex task that is difficult to compensate for, may also reveal subtle mobility deficits [24, 28, 51].Overall, there is no definitive way of objectively understanding the reasons for lack of differences in free-living gait quality between our cohorts of chronic mTBI patients and healthy controls. There are many unknown factors and contexts that affect free-living assessments. For example, here the environments participants were regularly walking in, the surfaces they walked on, or the types of terrain encountered were all unknown and such heterogeneity could impact results [52]. Equally, it is not possible to quantify the usual free-living mobility habits of the participants or to determine if this chronic mTBI cohort displayed any compensatory behaviour strategies (e.g., refraining from talking or performing other tasks whilst walking) that could further impact results. The introduction of egocentric video recordings of free-living mobility may enable greater insight and a robust reference to better understand the context of environments [53]. If used in conjunction with objective free-living IMU assessment, video data could yield even greater contextual understanding of free-living gait performance and any compensatory behaviour mTBI patients display within an environment.

### Strengths and limitations

Digital technologies such as IMU’s have many advantages over traditional methods of assessment including objectivity and continuous data collection. The primary strength of this study was the use of a single IMU to objectively measure free-living gait quality in chronic mTBI patients and controls; the use of a single device and assessment within usual daily life means that subjects had low research burden [54]. We also quantified useful gait quality metrics from clinical-based conceptual models from neurological-based research. Although use of a single IMU alone on the lower back facilitated more rapid data collection and reduced burden, it fails to quantify other useful gait characteristics which may provide more insight to dynamic postural control and environmental information i.e., step width and step width variability arising from uneven terrain [55].Thus, future research should investigate additional gait characteristics (based on conceptual gait models) with e.g., multiple IMU’s (on the feet) or a video-based wearable for a more informed free-living assessment. While the authors are not currently aware of any IMU-based technology to quantify step width during free-living, a computer vision approach has been suggested from a wearable camera [53]. Additionally, the outcome measures presented are primarily research-orientated, requiring a great deal of time-consuming post-processing and checking, which is based on prior experience of inertial data [56, 57]. Therefore, there are needs to refine and deploy software that clinicians and patients can easily navigate, which would allow more widespread uptake and use by health professionals [57].

No power calculation was used in this study as it was based as an exploratory study with opportunistic sampling. This may have limited the strength of any conclusions drawn and should be taken with caution. Future research should aim to utilise power calculations to ensure sufficient sample size and ability to detect small differences in results. Participants were assessed for ~ 7 days using a single IMU attached to a waist belt. However variation in the exact length of time participants wore wearables (minimum three days) could introduce differences and therefore not reflect true habitual free-living mobility as used in other studies [48, 58]. Using multiple IMUs may provide more detailed spatial and temporal data for turning, balance and gait as used in previous studies [24], but this carries different limitations; such as longer data download, processing complexity and increased wearer burden, limiting the practical or clinical application. This trade-off should be considered in future studies as a potential improvement to the assessment protocol. [59, 60].

There were some additional limitations to this study. First, a more detailed demographic profile could be reported in future studies to derive further inferences about the free-living mobility results or underlying physiological mechanisms for persistent symptom and mobility deficits [24]. For example, the symptom questionnaires were limited to NSI that were only completed by the mTBI cohort, which limited any useful comparisons and inference on the relationship between groups [3]. Second, balance problems in the chronic mTBI group were self-reported with no baseline or robust analysis done to quantify the magnitude of impairment [3], with the many factors such as the previous history of mTBI and evidence of abnormal neuroimaging omitted [4, 61]. Third, the differences in this mTBI cohort's chronicity are likely to limit the direct comparison with other studies. Our study's cohort was chronic with a median post-injury time greater than 1-year, which compared to other studies examining people post-mTBI is a longer time since injury [24, 62].

## Conclusions

Our results demonstrate that free-living IMU-based gait quality metrics were not significantly different between patients with chronic mTBI and healthy aged-matched controls. Despite a lack of significant findings herein, we feel that there is value in undertaking free-living mobility assessments. This study has highlighted that a single IMU can obtain a wealth of continuous free-living gait quality measures in people with symptomatic chronic mTBI and healthy controls. While this exploratory study indicated no between group differences, we feel that this work provides a foundation for future work in this area, where a-priori power and sample size are controlled. When considering the results of this study with our previous findings [3], we advocate that assessments of free-living mobility should include both measures of gait and turning quality. Future research should also focus on (i) additional gait characteristics from conceptual gait models and (ii) longitudinal analysis of chronic mTBI patients during different stages of recovery (acute to chronic) to holistically monitor mobility impairments and recovery. Improving objectivity in mTBI assessment will result in greater understanding of injury progression, recovery, and rehabilitation across a variety of clinical settings.

## Data Availability

De-identified data generated from this study will be deposited into the Federal Interagency Traumatic Brain Injury Research (FITBIR) Informatics System.
